# Uptake of COVID-19 Booster Dose among Saudi Arabian Population

**DOI:** 10.3390/medicina58070972

**Published:** 2022-07-21

**Authors:** Najim Z. Alshahrani, Abdullah A. Alsabaani, Iman Ridda, Harunor Rashid, Faris Alzahrani, Talal Hamed Almutairi, Bader Ahmed S. Alzahrani, Abdulelah Saleh Saeed Albeshri

**Affiliations:** 1 Department of Family and Community Medicine, Faculty of Medicine, University of Jeddah, Jeddah 21589, Saudi Arabia; 2 Department of Family and Community Medicine, College of Medicine, King Khalid University, Abha 62529, Saudi Arabia; aalsabaani@kku.edu.sa; 3 Department of Public Health, National University of Natural Medicine, Portland, OR 97201, USA; iridda@nunm.edu; 4 National Centre for Immunisation Research and Surveillance (NCIRS), Westmead, NSW 2145, Australia; harunor.rashid@health.nsw.gov.au; 5 Discipline of Child and Adolescent Health, The Children’s Hospital at Westmead Clinical School, Westmead, NSW 2145, Australia; 6 Marie Bashir Institute for Infectious Diseases and Biosecurity, School of Biological Sciences and Sydney Medical School, The University of Sydney, Westmead, NSW 2145, Australia; 7 Department of Public Health, General Directorate of Health Affairs in Aseer Region, Ministry of Health, Abha 62523, Saudi Arabia; fmaalz2012@hotmail.com; 8 College of Medicine, King Saud Bin Abdulaziz University for Health Sciences (KSAU-HS), Riyadh 14611, Saudi Arabia; t-h-a333@hotmail.com; 9 General Directorate of Health Affairs, Ministry of Health, Jeddah 23222, Saudi Arabia; Bader.ahmedsa@gmail.com; 10 College of Medicine, King Khalid University, Abha 62529, Saudi Arabia; 4341111dba@gmail.com

**Keywords:** COVID-19, COVID-19 vaccine, vaccination, COVID-19 vaccine booster, vaccine uptake, Saudi Arabia

## Abstract

*Backgroundand objectives*: Although several vaccines have been produced and administered around the world, new SARS-CoV-2 worsened the COVID-19 infection risk and impacted the initial vaccine dosage effectiveness. Based on studies indicating that the third and fourth COVID-19 vaccine doses significantly reduced COVID-19 transmission, Saudi Arabia has been administering COVID-19 booster vaccine doses to its citizens. The purpose of this study was to evaluate the uptake of the COVID-19 vaccine booster in relation to the socio-demographic characteristics and other associated factors among the Saudi population. *Materials and Methods*: This study was an online analytical cross-sectional study using a self-administered questionnaire. Pearson Chi-square test and multiple logistic regression analyses were used to determine factors associated with the uptake of COVID-19 booster dose vaccines. *Results*: A total of 2332 responded to our study. Overall, 527 (22.6%) participants had received a booster dose. An age of 55 and above (aOR: 5.415; 95% CI: 2.719–10.783), Eastern region (aOR: 2.513; 95% CI: 1.566–4.033), history of influenza vaccination at annual intervals (aOR: 2.387; 95% CI: 1.730–3.293), the first dose of Moderna vaccine (aOR: 1.324; 95% CI: 1.160–1.510), and cancer (aOR: 2.161; 95% CI: 1.218–3.879) were independent factors most associated with a higher uptake of the COVID-19 vaccine booster dose. In contrast, the second dose of Moderna vaccine (aOR: 0.794; 95% CI: 0.683–0.922), AstraZeneca vaccine (aOR: 0.691; 95% CI: 0.509–0.939), strong symptoms from side effects after the second dose of the COVID-19 vaccine (aOR: 0.615; 95% CI: 0.404–0.935) were independent factors most associated with a lower uptake of the COVID-19 vaccine booster dose. *Conclusions*: Our findings indicate low COVID-19 vaccine booster uptake. This necessitates the need for strategies to address discouraging factors of the COVID-19 vaccine booster dose uptake and engage the Saudi population to raise awareness about the importance of the booster dose.

## 1. Introduction

In December 2019, cases of atypical pneumonia due to an unknown cause were reported to the World Health Organization (WHO) from Wuhan, China [1,2]. The causative organism was later identified as a novel coronavirus formally named ‘severe acute respiratory syndrome coronavirus 2’ or ‘SARS-CoV-2’, and the disease was called ‘COVID-19′ [3]. Several vaccines have been produced and administered around the world since late 2020 [4], leading to the most extensive vaccination campaigns in history [5]. However, variants of SARS-CoV-2, such as B.1.1.7 strain, P.1, B.1.617 strain, and B.1.1.529 strain, worsened the COVID-19 infection risk and impacted the initial vaccine dosage effectiveness [6]. Studies had to be conducted and later indicated that the third and fourth COVID-19 vaccine doses significantly reduced COVID-19 transmission and severity [7], and increased the titers of antibodies against variants [1]. Therefore, the WHO has recommended COVID-19 booster vaccination for all people who have received their minimum essential primary doses [3]. Saudi Arabia commenced a booster vaccination program in December 2021 for individuals aged 16 years and above. However, vaccine hesitancy remains a major hindrance to achieving optimum vaccination coverage in the country. As of 14 February 2022, only 67.8% of the Saudi population were fully vaccinated [8]. There is considerable variation in vaccine acceptability and the existence of vaccine hesitancy not only in Saudi Arabia but also in other countries across the globe. For instance, in Europe, vaccine acceptability was reported in only a third of the population, in contrast to 85% of the population in South Asia [9]. A study from China showed that the COVID-19 vaccine booster acceptability rate was 76.8% [1], while in the United States of America (USA), it was even lower at 62% [4].

Immunization experts have carefully considered whether COVID-19 booster doses for specific susceptible groups or everyone are necessary to bolster immunity in order to combat the newly emerging strains of COVID-19 and there is uncertainty about the duration of protection offered by the vaccines [2]. As public awareness about the importance of booster doses grows, concerns regarding the safety of different vaccines increases. Depending on the information received, if an individual believed he or she was at higher risk of getting COVID-19 without a booster dose, a choice is easily made to take it up. This indicates that despite concerns, when benefits outweigh risks, people tend to accept the vaccine booster dose.

A study at Ajman University showed that the average knowledge score was 44.6% for the third COVID-19 booster dose, but the attitude score was 70.2% [10]. In this study, higher education level, healthcare employment, history of COVID-19 infection, and immunization against COVID-19 were associated with higher knowledge and better attitude scores, while in China, perceived benefits of booster dose, young age, and being employed were factors to higher acceptability rates [2]. Previous studies show that reasons for willingness to take a booster dose is based majorly on the information and communication regarding the benefits and efficacy of the booster doses [1,2,4,10,11]. However, in Saudi Arabia, there is a scarcity of studies exploring COVID-19 vaccine booster dose uptake among the general population. Factors influencing the COVID-19 booster vaccine uptake in Saudi Arabia are not studied. Therefore, our study’s purpose was to evaluate the uptake of the COVID-19 vaccine booster in relation to the socio-demographic characteristics and other associated factors among the Saudi population. Our study findings which will help the Ministry of Health (MOH) and other healthcare stakeholders to establish the best approach for promoting the booster dose uptake in the country.

## 2. Materials and Methods

### 2.1. Study Design

We conducted a cross-sectional study from December 2021 to March 2022. All adults aged 18 years and older who were vaccinated against COVID-19 and had taken the vaccine booster dose were eligible for this study. We used an anonymous and self-administered questionnaire designed following the examples of previous similar studies assessing COVID-19 vaccine acceptance and uptake [9,10,11,12,13,14,15,16]. Then, we adjusted it to suit our study participants. It was pilot tested on 30 non-selected individuals for validity. The questionnaire consisted of 5 parts: The first inquired about participants’ socio-economic and demographic characteristics and both influenza and COVID-19 vaccination history. The second assessed participants’ uptake of COVID-19 vaccine booster dose. The third assessed participants’ attitude toward COVID-19 vaccine booster dose. The fourth evaluated any association between COVID-19 vaccine booster dose uptake and compliance to COVID-19 preventive measures. The final part inquired about trustworthy sources of COVID-19 information and their relationship with vaccine booster uptake. Questionnaires were distributed via email and social media through Google form links. Responses from 3500 eligible respondents were considered for this study.

### 2.2. Measurements

The uptake of the COVID-19 vaccine booster dose was our study outcome variable. Participants were asked if they had received the vaccine booster dose and had to answer YES or NO.

The age variable was categorized into: 18–25, 26–35, 36–45, 46–54, and 55 and above years. Gender was binary: male or female, as was marital status: married or single. Residence was divided in five regions: Northern, Southern, Western, Eastern, and Central. Education level was divided into four levels: below secondary school, secondary school, bachelor’s, and postgraduate degrees. Employment status, healthcare work employment type, obesity status, and smoking were dichotomous, and responses were YES or NO. Monthly income in Saudi Riyal (SAR) was grouped into 3 categories: less than SAR 5000, SAR 5000–10,000, and more than SAR 10,000.

Participants were asked about vaccination history. Answering a question about influenza vaccine, they had to choose from “never got before”,” in irregular intervals”, or “annual intervals”. Answering a question about the first or second dose of COVID-19 vaccine, they had to choose from “Pfizer”, “Moderna”, “Oxford AstraZeneca”, or “other”. The respondents were asked if they contracted COVID-19 infection and had to answer YES or NO. Regarding when they got COVID-19 in relation to vaccination, they had to choose from “before the first dose”, “after the first dose”, or “after the second dose”.

Vaccine side effect symptoms or COVID-19 symptoms experienced were grouped into: no symptoms, mild symptoms (fever and chills and others that resolved without going to hospital), moderate symptoms (symptoms needing treatment taken at home), strong symptoms (patient admitted in the medical ward), and critical (ICU admission). Then, participants were asked to list the symptoms experienced.

On a three-point Likert scale (Disagree, Neutral, and Agree), participants gave answers to statements about their trust in vaccines, healthcare professionals, public health and government institutions, different reasons for them to get COVID-19 vaccines, preventive recommendations, and keeping up with new COVID-19 information.

Participants were asked about chronic diseases, and they had to mention which chronic disease they had if they responded “Yes”. Finally, to know about the sources of COVID-19 information, participants were asked to list their most common sources of information about COVID-19 and its vaccines.

### 2.3. Data Analysis

Data were extracted, revised, coded, and entered into IBM SPSS version 22 (SPSS, Inc., Chicago, IL, USA) statistical software. All statistical analysis was done using two-tailed tests, and a *p* value less than 0.05 was statistically significant. A descriptive analysis based on the frequency and percent distribution was conducted for all variables. Crosstabulation was used to compare the intention to take up COVID-19 vaccine booster dose among variables and groups. Relationships between variables were tested using Pearson Chi-square test and an exact probability test for small frequency distributions. Subsequently, all variables with a statistically significant association with the dependent variable (received booster dose) were included in the multiple predictive models based on the backward stepwise method with a *p*-value of <0.05 as an entry criterion and a *p*-value of >0.05 as an exclusion criterion. Multiple logistic regression analyses were used to estimate adjusted odds ratios (aOR) and their 95% confidence intervals (95% CI) for the association of getting the COVID-19 booster dose with the predictors.

### 2.4. Ethical Considerations

The Research Ethics Committee at Security Forces Hospital Program in Holy Capital (HAP-02-K-052) reviewed and approved this research project being conducted. The approval number is ECM#0460-190122.

## 3. Results

A total of 2332 people participated in this study. Of all the participants, the majority (49.1%) were aged 18–25 years, were not obese (88.0%), and 51% were males. Most (61.4%) were not married, had a bachelor’s degree (70.8%), and were not employed (57.7%). Most employed participants (14.5%) worked outside the healthcare sector (85.5%). Of all the participants, the majority (57.6%) earned less than SAR 5000 monthly (57.6%), lived in the Central region (39.0%), and were nonsmokers (81.9%) (Table 1).

Overall, 527 (22.6%) participants had received the booster dose. Older age was associated with a higher uptake of the COVID-19 booster vaccine, which increased with age. Those aged 55 years and more significantly (*p* < 0.01) got vaccinated more with the booster dose (47.5%) than those aged 18–25 years (13.9%) despite the latter being the most predominant age group. More males (26.5%) significantly received the booster dose (*p* < 0.01) than females (18.5%). Being married was associated with almost double take-ups of the booster dose (30.1%) (*p* < 0.01) than being single (17.5%). The booster dose was received significantly more by participants in the Eastern region (33.6%) (*p* < 0.01). A higher education level was associated with more booster doses, with those with postgraduate degrees (41.3%) (*p* < 0.01) receiving significantly more booster doses. The COVID-19 vaccine booster dose was received significantly more by healthcare workers (*p* < 0.01) and employed participants (*p* < 0.01) in general (31.7% and 33.1%, respectively). Taking up the booster dose significantly increased with the increase in monthly income (*p* < 0.01). Those who earned more than SAR 10,000 were more than twice as likely to receive the booster dose than those who earned less than SAR 5000 (34.8% vs. 15.6%). Obese participants and smokers were also significantly more likely to take up the booster dose (*p* < 0.01).

Regarding COVID-19 and infection vaccination history, participants with a history of previous influenza vaccines were significantly more likely to take up the COVID-19 vaccine booster dose (*p* < 0.01). Participants who took influenza on an annual basis had a higher booster dose uptake rate (38.6%). The types of the vaccine on the first dose (*p* < 0.01) and the second dose (*p* = 0.002) were significantly associated with the uptake of the booster dose. Participants who took the first or second doses of the Moderna COVID-19 vaccines had the lowest booster dose uptake rates (5.0% and 8.0%, respectively). However, 71.8% and 77.4% received Pfizer vaccines as the first and second doses, respectively. Experiencing side effects on the second dose (*p* < 0.01), being infected with COVID-19 (*p* = 0.006), COVID-19 symptom severity (*p* < 0.01), having COVID-19 after receiving both doses (*p* < 0.01), and types of symptoms experienced (*p* = 0.028) were significantly associated with lower COVID-19 booster dose uptake. Participants who reported experiencing adverse effects following their second doses, and those who faced critical symptoms (admitted to ICU) were the least vaccinated with booster doses (16.7% and 12.5%, respectively). However, the difference was not statistically significant for the first dose receivers (*p* = 0.34).

**Figure 1:**
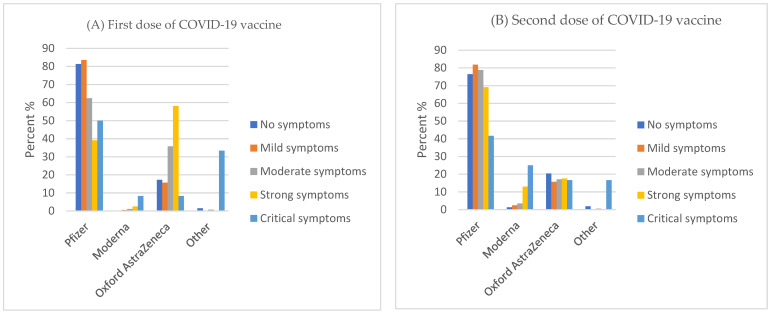
First dose (**A**) and second dose (**B**) of COVID-19 vaccines and their side effect symptom severity.

Symptom severity was negatively correlated with the increase in adverse effect cases among Pfizer vaccine first (Figure 1A) and second dose takers, as well as AstraZeneca vaccine second dose takers (Figure 1B). Among participants who took the first dose of Moderna and AstraZeneca vaccines, side effect symptoms remained steadily the same. On the other hand, Moderna’s second dose side effects were slightly positively correlated with the severity of symptoms.

Participants who had the COVID-19 infection after receiving a double dose of COVID-19 and reported critical symptoms were vaccinated least with booster doses (29.1% and 28.6%, respectively) (Table 2). In terms of COVID-19 symptoms, shortness of breath was associated with the highest booster uptake rate (19.8%), followed by anosmia (18.2%), myalgia (17.9%), and fatigue (17.6%).

Most participants who did not receive a booster dose had lost trust in the Saudi Ministry of Health (82.2%), WHO (85.3%), healthcare practitioners (82.1%), the effectiveness of all vaccines (85.7%), COVID-19 vaccines (84.3%), and were not satisfied with the previous COVID-19 vaccine doses taken (84.2%). Overall, loss of trust in healthcare and public health professionals and institutions was significantly associated with less booster uptake (*p* < 0.01). However, practicing preventive measures and following up with COVID-19 news did not substantially impact the booster uptake (*p* > 0.05) (Table 3).

Having comorbidities (n = 501) was significantly associated with booster uptake (p < 0.01), with the most uptake (53.8%) in cancer patients, while the least was in patients with immune deficiency diseases (Table 4).

Multiple logistic regression analysis showed that age, residence region, being employed, previous influenza vaccination, type of COVID-19 vaccine received, side effect strong symptoms, faith in COVID-19 vaccine, and having cancer remained significant predictors of COVID-19 vaccine booster dose uptake (*p* < 0.05) (Table 5).

Participants aged 55 and above were three times more likely to get a booster dose than those aged 26–35 years (aOR: 5.415; 95% CI: 2.719–10.783). Living in other regions other than the East and West were not significantly associated with the booster dose uptake. However, living in the East was almost two times more associated with the booster dose uptake (aOR: 2.513; 95% CI: 1.566–4.033). Influenza vaccination at annual intervals was two times more associated with more vaccine booster doses (aOR: 2.387; 95% CI: 1.730–3.293) than irregular influenza vaccination. Although the first dose of the Moderna vaccine was associated with more booster dose uptake (aOR: 1.324; 95% CI: 1.160–1.510), the second dose of the Moderna vaccine was associated with about 21% less booster dose uptake (aOR: 0.794; 95% CI: 0.683–0.922), while the second dose of AstraZeneca vaccine was associated with 30% less booster dose uptake (aOR: 0.691; 95% CI: 0.509–0.939). On the other hand, participants who did not lose or have faith in COVID-19 were also more likely to get booster doses (aOR: 2.426; 95% CI: 1.207–4.863). Participants who had cancer were two times more likely to get booster vaccine doses than those without cancer (aOR: 2.161; 95% CI: 1.218–3.879).

Even if it is expected that the worse their side effect symptoms are, the less people are less likely to take up the booster dose, participants who experienced critical symptoms were 19% less likely to get a booster dose (aOR: 0.814; 95% CI: 0.720–0.920), while those with strong and moderate symptoms were 39% (aOR: 0.615; 95% CI: 0.404–0.935) and 29% (aOR: 0.710; 95% CI: 0.507–0.9951) less likely to get a booster dose, respectively. Other variables analyzed were not statistically significant predictors.

Regarding the associations between COVID-19 vaccine booster uptake rate and agreement with trusted sources of COVID-19 information for participants, there was statistical significance across all the parameters (*p* = 0.000). However, those who followed scientifically developed resources, such as articles, health practitioners, the WHO, and the Ministry of Health, had the highest uptake of booster doses. In contrast, those who got information from social media, celebrities, influencers, friends, and family had the lowest COVID-19 vaccine booster uptake (Table 6).

## 4. Discussion

This study evaluated the acceptability and uptake of booster doses of the COVID-19 vaccine in relation to the socio-demographic and other associated factors in Saudi Arabia. Several studies [1,2,3,4,10,11,12,13,14,15,17] that explored the acceptability of a booster dose of the COVID-19 vaccine exist across the globe. However, this study was the first conducted in Saudi Arabia comparing COVID-19 booster takers and non-takers. All of our study participants received the first and second doses of COVID-19 vaccines, and some of them had received the booster vaccine dose. The majority of these participants were males, married, aged between 18 and 25 years, and employed. These findings align with other studies that reported the young age and male groups to be predominant [12,13].

Although some evidence showed that more elderly people should have received the booster doses than the young adults [14], our findings contrasted it by showing that more young adults aged 18–25 years received the booster doses. These results are in line with a study that was conducted in Hong Kong which showed only 31.6% of community-dwelling older adults aged ≥65 years had received a COVID-19 vaccine booster dose [17]. However, another study that was conducted in adult factory workers showed that 84% intended to receive a free booster dose of the COVID-19 vaccine within the next six months [18,19]. Usually, younger adults are more hesitant till they are convinced of the vaccine’s safety, and they often think that they are not high risk enough to consider getting vaccinated [15]. In our study, although this observation might not necessarily be explained by the individuals’ belief in vaccine safety, the percentage of young adults across the country could explain it. Two-thirds of Saudi Arabia’s population is below 29 years old [16]; hence, a higher percentage response rate in this study is from young people.

Married couples are expected to be more interested in vaccination than unmarried people because of their responsibilities to protect and take care of their children and spouses. Similarly, the males, being the heads of most of the families in Saudi Arabia, are expected to lead by example and also have responsibilities to protect their families from diseases, which might have made more of them accept the booster dose. The working class in Saudi Arabia also consists of more males than females, which might make them more receptive to the COVID-19 vaccines to be able to access workplaces. Most of the employed participants received the booster dose. The reason for that might be the vaccine mandates placed by different public and private institutions to ensure safe workplaces [18]. On 7 May 2021, the Saudi Ministry of Human Resources and Social Development (MHRSD) mandated that only vaccinated workers had to attend the workplace. This mandate was enforced on 1 August 2021 by the ministry of interior (MoI). We also found that working in the healthcare sector was associated with higher COVID-19 booster uptake than working in other sectors, as expected since healthcare workers have a better understanding of the vaccines. These findings agree with other previous studies [4,12].

High income and high education levels were positively correlated with COVID-19 vaccine booster acceptability, where those who earned more than SAR 10,000 were more than twice as likely to receive the booster dose than those who earned less than SAR 5000 (34.8% vs. 15.6%), and uptake of the COVID-19 booster dose increased as the education levels increased. This can be explained by the fact that most high-income earners are leaders in different sectors who had to be exemplary to other workers by getting vaccinated earlier, which made them qualify for a booster dose earlier too. That is also in line with education’s impact on the take up of the COVID-19 booster dose. Highly educated people usually occupy high-income job positions and are more likely to be better informed on vaccines as their sources of information are more likely to be genuine, such as scientific papers and healthcare and public health professionals’ contacts. Some studies have reported similar findings as ours, and in addition, it has been reported that higher education institutions were more likely to impose vaccine mandates [20]. This might explain why we found that participants with postgraduate studies were the most vaccinated with the booster dose of COVID-19 vaccines. Similar findings of a positive correlation of education with booster dose uptake were also reported by Yadete et al. [4] and Folcarelli et al. [12].

More participants who had obesity and who were smokers received booster doses than others. COVID-19 was found to be more severe in individuals with chronic conditions and obesity, and the COVID-19 vaccine booster dose is recommended more in people with comorbidities. Obesity and smoking are known risk factors for most chronic diseases, and most participants who were smokers might have other chronic diseases. Therefore, these conditions might have encouraged them to receive the booster doses [14].

Our findings also indicated that the majority of the participants received Pfizer vaccines, followed by Oxford AstraZeneca. The efficacy of these two vaccines is believed to be the same with similar side effects. In Saudi Arabia, Pfizer vaccines were distributed more than any other vaccine type. Generally, the Pfizer and AstraZeneca vaccine side effects were mostly mild. We found a decrease in side effects as the severity increased for Pfizer and AstraZeneca vaccines, with more severe side effects reported after the second dose. Side effects for Moderna vaccines were mostly the same throughout. Previous studies have reported reduced side effects for the Pfizer vaccine compared to Moderna vaccines [21]. On the other hand, a study from the UAE comparing the Pfizer-BioNTech vaccine and Sinopharm vaccines found more side effects for the Pfizer-BioNTech vaccine [22]. This difference might be due to the technology used in these vaccines. The Pfizer vaccine is mRNA based, while Sinopharm is based on the inactivated virus. In line with our findings, another study conducted in the USA found that the Moderna vaccine had relatively more side effects, especially after the second dose, than the Pfizer and Johnson & Johnson vaccine [23]. This might explain why we found that participants who took first or second doses of Moderna COVID-19 vaccines experienced adverse effects following their second doses or faced critical symptoms.

A history of taking annual influenza vaccine was associated with a high uptake of COVID-19 booster vaccines. This might be because participants who take regular influenza vaccines are well informed about vaccines and perceive them as safe since they are used to them. In a study carried out in the UAE, they found similar influenza vaccine uptake as a factor for acceptance of COVID-19 vaccines [24].

We found that participants who had COVID-19 infection after receiving a double dose of COVID-19 and who reported critical symptoms were the least vaccinated with booster doses. Several studies have shown that COVID-19 vaccines reduce the likelihood of infection and COVID-19 severity [10,16,25]. Being infected with COVID-19 after being fully vaccinated might have discouraged them and increased mistrust of the vaccine leading to a lower uptake of the booster dose. There are several factors that could have led to the reinfection, including COVID-19 new variants, chronic diseases, obesity, and the elderly, as indicated by previous studies [25,26].

This study showed that adverse effect symptoms significantly influence the booster uptake, with shortness of breath being associated with the highest booster uptake rate, followed by anosmia, myalgia, and fatigue. These findings are similar to what was found in a study conducted by Meo et al. [21], who found them more among people vaccinated with the Moderna vaccine, further supporting why the Moderna vaccine was associated with less booster uptake as found in our study.

Overall, loss of trust in healthcare and public health professionals and institutions was significantly associated with less booster uptake. This might be caused by the frequent changes in the scientific information that comes out as science uncovers more about COVID-19, which can lead to mistrust in science, scientists, scientific institutions, and healthcare professionals and institutions. Most participants who did not receive a booster dose were not satisfied with the previous COVID-19 vaccine doses taken and lost trust in the Saudi Ministry of Health, WHO, healthcare practitioners, and the effectiveness of all vaccines. This can be explained by the possibility of reinfection after being fully vaccinated, which is contrary to what they thought before taking vaccines when they thought that it was completely protective against COVID-19. Strategies to approach them, more education programs focusing on different reasons why vaccines are not 100% protective, such as the new variants, and the effectiveness percentage of each vaccine, as well as education on the importance of a booster dose and COVID-19 vaccination in general, are needed. Our findings are also supported by other previous studies indicating that mistrust of science and health institutions could impede preventive measures and vaccination [27,28]. This highlights the need for educating the people about vaccine efficacy and safety and addressing their misconceptions and beliefs by supporting open communication [14].

Comorbidities, such as diabetes, hypertension, cancer, respiratory disease, asthma, and renal and immunodeficiency diseases, were positively correlated with receiving booster doses. Cancer was associated with the highest booster vaccine uptake, followed by diabetes. People with chronic diseases have been encouraged to get vaccinated to lower their increased risk of mortality and morbidity from COVID-19, and it was found that people with higher perceived risks demonstrated lower vaccination hesitancy [14]. This might explain the higher uptake of the COVID-19 booster dose among our study participants with chronic diseases. Vulnerable groups included people with chronic diseases, and they were the first to be vaccinated at the start of the vaccination campaigns against COVID-19. Therefore, the earlier they were vaccinated, the earlier they fulfilled the requirements for the booster dose and the more they are represented among the COVID-19 booster dose receivers.

Vaccinated people have been gradually increasing as time goes on. In Saudi Arabia, data indicate that less than 5% of people were fully vaccinated in July 2021 [29]. However, in the third quarter of 2021, the fully vaccinated people rose from 5% to at least 50% [14]. This rise in vaccination uptake peaked at about 70% in the fourth and last quarter of 2021 [29], mainly due to strategies adopted to improve the knowledge and awareness regarding the significance of COVID-19 vaccines.

Multiple logistic regression analysis showed that age, residence region, being employed, previous influenza vaccination, type of COVID-19 vaccine received, side effect strong symptoms, faith in COVID-19 vaccine and having cancer remained significant independent predictors of COVID-19 vaccine booster dose uptake. This confirms that these factors had strong associations with the uptake of COVID-19 booster vaccine doses. These results are similar to comparative results we discussed above. However, logistic regression showed that participants who regularly tool influenza vaccine were two times more likely to take up COVID-19 booster doses than the ones who tool it irregularly. This is expected since people with a history of irregularities are more likely to keep up with COVID-19 vaccination schedules. Living in the Eastern region was two times more associated with the booster uptake than living in the Western region. The first cases of COVID-19 in Saudi Arabia were discovered in the East, which might have led to more efforts to combat the pandemic and more awareness among the residents and subsequently more and earlier uptake of vaccines [28]. The earlier they took the first and second doses, the earlier they took booster doses and the more they are dominant in our sample.

We found that participants who followed scientifically developed resources such as scientific articles, health practitioners, the WHO, and the Ministry of Health had a higher uptake of booster doses compared to those who got information from social media, celebrities, influencers, friends, and family. This indicates that trusted sources of COVID-19 information are scientific articles, health practitioners, the WHO, and the Ministry of Health. Previous studies have indicated that social media and other unofficial sources of information were associated with more conspiracy theories about COVID-19 and vaccines, which could explain the lower uptake rates among participants who use them as their sources of information [30,31]. Zhang et al. found that among Chinese workers, social media information about COVID-19 from friends influence the behavior to take up the vaccine boosters [19]. It was found that misinformation about COVID-19 circulated faster among friends and families through social media. This includes false prevention, conspiracies, and denial of COVID-19’s existence [31]. This prompted the WHO to create social media-friendly infographics to debunk myths. With the increasing use of social media and other sources of information, feeding them with accurate information from those trusted sources would increase the booster acceptability and help accurate health information to reach more people who are more likely not to get the official and scientific sources of information. This is supported by a previous study that found that sharing trustworthy information through social media by healthcare professionals and institutions, partnering with social media platforms, and establishing a team assigned to debunk misinformation and share accurate information using social media was more effective than trying to direct the general population to institutional websites and scientific publications [31,32,33]. In addition, the uptake of the vaccine booster dose can be increased by a collaboration between the public and private sectors, enabling Saudi Arabia to reach herd immunity quickly. The findings from the present study could help the healthcare organizations, government, and other authorities to design strategies that augment the public’s knowledge, awareness, and perception of the COVID-19 vaccine booster dose.

The quantitative study design used limited the exploration of some aspects in detail, rather than in a structured format. A qualitative study design alongside the quantitative one would generate more information about COVID-19 vaccine booster doses. The convenience sampling method and online nature of this study cause under- or over-representation of the Saudi population and are prone to bias. Therefore, extensive experimental or longitudinal studies would mitigate these limitations. Since more people are becoming eligible for a vaccination with the COVID-19 vaccine booster dose every day, the results presented in this study might not accurately represent the current situation. We recommend further studies to remain up to date.

## 5. Conclusions

This study showed that older age, male gender, being married, higher education levels, high income, previous influenza vaccine, having comorbidities, consulting the Ministry of Health, the WHO, healthcare professions, and scientific articles as sources of information were associated with a high uptake of the COVID-19 booster dose. In contrast, being vaccinated with Moderna COVID-19 vaccines, experiencing side effects on the second dose and critical symptoms, infection with COVID-19 after full vaccination, and loss of trust in healthcare, public health professionals and institution, and using social media, celebrities, influencers, friends, and family as sources of information were associated with a lower uptake of the COVID-19 booster vaccines. We recommend healthcare organizations, the government, and other authorities design strategies that augment the public’s knowledge, awareness, perception, and debunk myths about the COVID-19 vaccine booster dose. Extensive longitudinal and offline studies with larger samples are also recommended to explore, in detail, the booster dose uptake.

## Figures and Tables

**Table 1:** Socio-demographic characteristics of the study’s respondents.

		Got COVID-19 Booster Dose						
		No (*n* = 1805)		Yes (*n* = 527)		Total (*n* = 2332)		*p*-Value
		*N*	%	*N*	%	*N*	%	
Age in years	18–25	986	(86.1%)	159	(13.9%)	1145	49.1%	<0.01 *
	26–35	457	(73.2%)	167	(26.8%)	624	26.8%	
	36–45	227	(65.8%)	118	(34.2%)	345	14.8%	
	46–54	103	(65.6%)	54	(34.4%)	157	6.7%	
	55 and more	32	(52.5%)	29	(47.5%)	61	2.6%	
Gender	Male	874	(73.5%)	315	(26.5%)	1189	51.0%	<0.01 *
	Female	931	(81.5%)	212	(18.5%)	1143	49.0%	
Marital status	Single	1181	(82.5%)	250	(17.5%)	1431	61.4%	<0.01 *
	Married	624	(69.3%)	277	(30.7%)	901	38.6%	
Region of living	Northern	241	(87.6%)	34	(12.4%)	275	11.8%	<0.01 *
	Southern	440	(77.6%)	127	(22.4%)	567	24.3%	
	Eastern	180	(66.4%)	91	(33.6%)	271	11.6%	
	Western	223	(71.9%)	87	(28.1%)	310	13.3%	
	Central	721	(79.3%)	188	(20.7%)	909	39.0%	
Education	Below secondary	35	(68.6%)	16	(31.4%)	51	2.2%	<0.01 *
	Secondary	361	(79.0%)	96	(21.0%)	457	19.6%	
	Bachelor’s degree	1308	(79.2%)	344	(20.8%)	1652	70.8%	
	Postgraduate degree	101	(58.7%)	71	(41.3%)	172	7.4%	
Employment	No	1145	(85.1%)	201	(14.9%)	1346	57.7%	<0.01 *
	Yes	660	(66.9%)	326	(33.1%)	986	42.3%	
Health worker	No	1574	(78.9%)	420	(21.1%)	1994	85.5%	<0.01 *
	Yes	231	(68.3%)	107	(31.7%)	338	14.5%	
Monthly income	Less than SAR 5000	1135	(84.4%)	209	(15.6%)	1344	57.6%	<0.01 *
	SAR 5000–10,000	340	(70.5%)	142	(29.5%)	482	20.7%	
	More than SAR 10,000	330	(65.2%)	176	(34.8%)	506	21.7%	
Obesity	No	1606	(78.3%)	446	(21.7%)	2052	88.0%	0.007 *
	Yes	199	(71.1%)	81	(28.9%)	280	12.0%	
Smoker	No	1505	(78.8%)	404	(21.2%)	1909	81.9%	<0.01 *
	Yes	300	(70.9%)	123	(29.1%)	423	18.1%	

SAR: Saudi Riyal; 1 USD = 3.75 SAR; * statistically significant (*p* < 0.05).

**Table 2:** Vaccine history and infection history among study’s respondents.

		Got COVID-19 Booster Dose						
		No (*n* = 1805)		Yes (*n* = 527)		Total		*p*-Value
		*N*	%	*N*	%	*N*	%	
Influenza Vaccine	Never got before	859	81.5%	195	18.5%	1054	45.2%	<0.01 *
	In irregular intervals	776	77.5%	225	22.5%	1001	42.9%	
	Annual intervals	170	61.4%	107	38.6%	277	11.9%	
Type of vaccines for first dose	Pfizer	1341	80.1%	334	19.9%	1675	71.8%	<0.01 *
	Moderna	19	95.0%	1	5.0%	20	0.9%	
	Oxford AstraZeneca	430	69.6%	188	30.4%	618	26.5%	
	Other	15	78.9%	4	21.1%	19	0.8%	
Type of vaccines for 2nd dose	Pfizer	1383	76.6%	423	23.4%	1806	77.4%	<0.01 *
	Moderna	92	92.0%	8	8.0%	100	4.3%	
	Oxford AstraZeneca	313	76.9%	94	23.1%	407	17.5%	
	Other	17	89.5%	2	10.5%	19	0.8%	
Side effect after first dose	No symptoms	407	74.7%	138	25.3%	545	23.4%	0.34
	Mild symptoms	723	79.0%	192	21.0%	915	39.2%	
	Moderate symptoms	413	76.5%	127	23.5%	540	23.2%	
	Strong symptoms	252	78.8%	68	21.3%	320	13.7%	
	Critical symptoms	10	83.3%	2	16.7%	12	0.5%	
Side effect after second dose	No symptoms	386	72.8%	144	27.2%	530	22.7%	<0.01 *
	Mild symptoms	578	74.4%	199	25.6%	777	33.3%	
	Moderate symptoms	527	80.5%	128	19.5%	655	28.1%	
	Strong symptoms	293	84.7%	53	15.3%	346	14.8%	
	Critical symptoms	21	87.5%	3	12.5%	24	1.0%	
Infected with COVID-19	No	1370	76.1%	430	23.9%	1800	77.2%	0.006 *
	Yes	435	81.8%	97	18.2%	532	22.8%	
Onset of infection	Before the first dose	300	85.7%	50	14.3%	350	66.7%	<0.01*
	After the first dose	31	91.2%	3	8.8%	34	6.5%	
	After the second dose	100	70.9%	41	29.1%	141	26.9%	
Infection severity	No symptoms	39	73.6%	14	26.4%	53	9.9%	<0.01 *
	mild symptoms	80	79.2%	21	20.8%	101	18.9%	
	moderate symptoms	172	84.3%	32	15.7%	204	38.3%	
	Strong symptoms	140	83.3%	28	16.7%	168	31.5%	
	critical symptoms	5	71.4%	2	28.6%	7	1.3%	
Symptoms	Fever	327	83.2%	66	16.8%	393	75.0%	0.028 *
	cough	246	84.2%	46	15.8%	292	55.7%	
	SOB **	142	80.2%	35	19.8%	177	33.8%	
	Fatigue	361	82.4%	77	17.6%	438	83.6%	
	Myalgia	325	82.1%	71	17.9%	396	75.6%	
	Headache	347	83.4%	69	16.6%	416	79.4%	
	Anosmia	323	81.8%	72	18.2%	395	75.4%	
	Pharyngitis	236	84.3%	44	15.7%	280	53.4%	
	Congestion	243	83.5%	48	16.5%	291	55.5%	
	Rhinitis	165	83.8%	32	16.2%	197	37.6%	
	Nausea	185	85.6%	31	14.4%	216	41.2%	
	Diarrhea	145	84.3%	27	15.7%	172	32.8%	
	Vomiting	75	84.3%	14	15.7%	89	17.0%	

* Statistically significant (*p* < 0.05). ** Shortness of breath.

**Table 3:** Influencing factors of COVID-19 booster dose uptake.

		Got COVID-19 Booster Dose						
		No (*n* = 1805)		Yes (*n* = 527)		Total (*n* = 2332)		*p*-Value
		*N*	%	*N*	%	*N*	%	
I lost faith in the Saudi Ministry of Health	Disagree	708	77.5%	206	22.5%	914	39.2%	<0.01 *
	Agree	582	82.2%	126	17.8%	708	30.4%	
	Neutral	515	72.5%	195	27.5%	710	30.4%	
I lost faith in the World Health Organization	Disagree	1023	73.4%	370	26.6%	1393	59.7%	<0.01 *
	Agree	320	85.3%	55	14.7%	375	16.1%	
	Neutral	462	81.9%	102	18.1%	564	24.2%	
I lost my trust in healthcare practitioners	Disagree	1094	74.4%	377	25.6%	1471	63.1%	<0.01 *
	Agree	275	82.1%	60	17.9%	335	14.4%	
	Neutral	436	82.9%	90	17.1%	526	22.6%	
I lost faith in the effectiveness of all vaccines “All vaccines are useless”	Disagree	989	73.9%	349	26.1%	1338	57.4%	<0.01 *
	Agree	365	85.7%	61	14.3%	426	18.3%	
	Neutral	451	79.4%	117	20.6%	568	24.4%	
I lost faith in COVID-19 vaccines	Disagree	988	74.1%	346	25.9%	1334	57.2%	<0.01 *
	Agree	380	84.3%	71	15.7%	451	19.3%	
	Neutral	437	79.9%	110	20.1%	547	23.5%	
I was not satisfied with the previous doses	Disagree	978	74.4%	337	25.6%	1315	56.4%	<0.01 *
	Agree	443	84.2%	83	15.8%	526	22.6%	
	Neutral	384	78.2%	107	21.8%	491	21.1%	
Traveling forced me to get vaccinated (Health Passport)	Disagree	1022	75.8%	327	24.2%	1349	57.8%	0.084
	Agree	413	79.9%	104	20.1%	517	22.2%	
	Neutral	370	79.4%	96	20.6%	466	20.0%	
The desire to shop and go to malls forced me to get vaccinated	Disagree	944	75.2%	311	24.8%	1255	53.8%	0.020 *
	Agree	531	80.6%	128	19.4%	659	28.3%	
	Neutral	330	78.9%	88	21.1%	418	17.9%	
Entertainment activities forced me to get vaccinated	Disagree	1002	75.6%	324	24.4%	1326	56.9%	0.048 *
	Agree	442	79.4%	115	20.6%	557	23.9%	
	Neutral	361	80.4%	88	19.6%	449	19.3%	
I do not wear a mask anymore	Disagree	1016	75.9%	322	24.1%	1338	57.4%	0.12
	Agree	427	80.1%	106	19.9%	533	22.9%	
	Neutral	362	78.5%	99	21.5%	461	19.8%	
I no longer sanitize hands	Disagree	923	75.7%	296	24.3%	1219	52.3%	0.069
	Agree	491	80.5%	119	19.5%	610	26.2%	
	Neutral	391	77.7%	112	22.3%	503	21.6%	
I am no longer afraid of COVID-19 and its variants	Disagree	844	77.1%	250	22.9%	1094	46.9%	0.63
	Agree	540	78.6%	147	21.4%	687	29.5%	
	Neutral	421	76.4%	130	23.6%	551	23.6%	
I do not follow the news of COVID-19 in my country	Disagree	903	76.2%	282	23.8%	1185	50.8%	0.167
	Agree	491	80.1%	122	19.9%	613	26.3%	
	Neutral	411	77.0%	123	23.0%	534	22.9%	
I do not follow the news of COVID-19 in the world	Disagree	836	75.5%	272	24.5%	1108	47.5%	0.038 *
	Agree	550	80.6%	132	19.4%	682	29.2%	
	Neutral	419	77.3%	123	22.7%	542	23.2%	

* Statistically significant (*p* < 0.05).

**Table 4:** Association of COVID-19 Booster Dose Uptake with Participants Who Have Chronic Diseases.

Chronic Disease	Got COVID-19 Booster Dose					
	No		Yes		Total (*N* = 501)	
	*N*	%	*N*	%	*N*	*p*-Value
DM	55	53.40%	48	46.60%	103	<0.01 *
HTN	64	62.70%	38	37.30%	102	
Cancer	6	46.20%	7	53.80%	13	
CVS	24	63.20%	14	36.80%	38	
Respiratory **	19	63.30%	11	36.70%	30	
Asthma	125	77.60%	36	22.40%	161	
Renal ***	13	65.00%	7	35.00%	20	
Immune deficiency disease	27	79.40%	7	20.60%	34	

* Statistically significant (*p* < 0.05). ** Include: asthma, COPD, tuberculosis, and emphysema. *** Glomerulonephritis, renal stones, chronic kidney disease, and renal failure.

**Table 5:** Multiple logistic regression analysis for estimates of predictors associated with getting COVID-19 vaccine booster dose.

	*p*-Value	aOR	95% C.I.	
			Lower	Upper
Age in years				
18–29 (ref)				
26–35	0.001 *	1.745	1.269	2.4
36–45	<0.01 *	2.478	1.621	3.787
46–54	<0.01 *	2.699	1.609	4.525
55 and more	<0.01 *	5.415	2.719	10.783
Gender				
Female (ref)				
Male	0.644	0.941	0.728	1.217
Marital status				
Unmarried (ref)				
Married	0.403	0.875	0.639	1.197
Region of living				
Northern (ref)				
Southern	0.087	1.484	0.944	2.333
Eastern	<0.01 *	2.513	1.566	4.033
Western	0.004 *	1.997	1.252	3.184
Central	0.06	1.495	0.983	2.272
Education				
Below secondary (ref)				
Secondary	0.567	0.816	0.406	1.638
Bachelor’s degree	0.435	0.765	0.39	1.5
Postgraduates’ studies	0.564	1.247	0.589	2.64
Employment				
No (ref)				
Yes	<0.01 *	1.729	1.281	2.332
Health worker				
No (ref)				
Yes	0.521	1.104	0.816	1.493
Monthly income				
less than SAR 5000 (ref)				
Monthly income SAR 5000–10,000	0.675	1.072	0.773	1.488
Monthly income more than SAR 10,000	0.908	1.022	0.704	1.484
Obesity				
No (ref)				
Yes	0.161	1.247	0.916	1.698
Smoker				
No (ref)				
Yes	0.474	1.109	0.835	1.472
Influenza Vaccine				
“Never got before (ref)”				
“In irregular intervals”	0.003 *	1.437	1.134	1.821
“Annual intervals”	<0.01 *	2.387	1.73	3.293
Type of vaccines for 1st dose				
“Pfizer” (ref)				
“Moderna”	<0.01 *	1.324	1.16	1.51
“Oxford AstraZeneca”	0.383	0.381	0.045	0.304
“Other”	0.105	8.676	0.634	18.656
Type of vaccines for 2nd dose				
“Pfizer” (ref)				
“Moderna”	0.003 *	0.794	0.683	0.922
“Oxford AstraZeneca”	0.018 *	0.691	0.509	0.939
“Other”	0.077	0.066	0.003	1.348
Side effect after 1st dose				
“No symptoms” (ref)				
“Mild symptoms”	0.758	1.02	0.898	1.158
“Moderate symptoms”	0.257	0.965	0.712	1.309
“Strong symptoms”	0.725	0.927	0.609	1.411
“Critical symptoms”	0.942	1.083	0.124	9.419
Side effect after 2nd dose				
“No symptoms” (ref)				
“Mild symptoms”	0.877	0.976	0.723	1.318
“Moderate symptoms”	0.046 *	0.71	0.507	0.9951
“Strong symptoms”	0.023 *	0.615	0.404	0.935
“Critical symptoms”	0.001 *	0.814	0.72	0.92
Infected with COVID-19				
“No” (ref)				
“Yes”	0.471	0.462	0.057	3.773
I lost faith in the Saudi Ministry of Health				
“Disagree” (ref)				
“Agree”	0.748	0.755	0.137	4.176
“Natural”	0.471	0.462	0.057	3.773
I lost faith in COVID-19 vaccines				
“Disagree” (ref)				
“Agree”	0.31	1.4	0.7334	2.685
“Neutral”	0.013 *	2.426	1.207	4.863
Cancer patient				
No (ref)				
Yes	0.008 *	2.161	1.218	3.879

aOR = adjusted odds ratio. C.I. = confidence interval. * Statistically significant (*p* < 0.05). (ref) = reference.

**Table 6:** Source of information related to COVID-19 and booster dose among study participants.

	Got COVID-19 Booster Dose *					
	No		Yes		Total	
	*n*	%	*n*	%	*n*	%
Health practitioners	364	72.50%	138	27.50%	502	65.50%
World Health Organization	311	71.30%	125	28.70%	436	56.90%
Ministry of health	499	72.40%	190	27.60%	689	89.90%
Research articles	350	71.00%	143	29.00%	493	64.40%
Books	202	72.70%	76	27.30%	278	36.30%
The schools and universities	189	74.10%	66	25.90%	255	33.30%
Celebrity and Influencers	46	75.40%	15	24.60%	61	8.00%
Friends	148	77.50%	43	22.50%	191	24.90%
Family	183	75.30%	60	24.70%	243	31.70%
Internet	289	79.20%	76	20.80%	365	47.70%
Social media	224	78.90%	60	21.10%	284	37.10%
TV	226	72.00%	88	28.00%	314	41.00%
Radio	111	72.10%	43	27.90%	154	20.10%
Mosque and religious pulpits	147	73.50%	53	26.50%	200	26.10%

* All significant (*p*-value < 0.05).

## Data Availability

Data available upon request.
